# Cross-Modal and Contrastive Optimization for Explainable Multimodal Recognition of Predatory and Parasitic Insects

**DOI:** 10.3390/insects16121187

**Published:** 2025-11-22

**Authors:** Mingyu Liu, Liuxin Wang, Ruihao Jia, Shiyu Ji, Yalin Wu, Yuxin Wu, Luozehan Xie, Min Dong

**Affiliations:** 1 China Agricultural University, Beijing 100083, China; 2 National School of Development, Peking University, Beijing 100871, China

**Keywords:** insect pest management, natural enemy recognition, multimodal deep learning, cross-species contrastive learning, ecological monitoring and interpretation

## Abstract

Natural enemies play a vital role in pest suppression and ecological balance within agricultural ecosystems. However, conventional vision-based recognition methods are highly susceptible to illumination variation, occlusion, and background noise in complex field environments, making it difficult to accurately distinguish morphologically similar species. To address these challenges, a multimodal natural enemy recognition and ecological interpretation framework, termed MAVC-XAI, is proposed to enhance recognition accuracy and ecological interpretability in real-world agricultural scenarios. The framework employs a dual-branch spatiotemporal feature extraction network for deep modeling of both visual and acoustic signals, introduces a cross-modal sampling attention mechanism for dynamic inter-modality alignment, and incorporates cross-species contrastive learning to optimize inter-class feature boundaries. Additionally, an explainable generation module is designed to provide ecological visualizations of the model’s decision-making process in both visual and acoustic domains. Experiments conducted on multimodal datasets collected across multiple agricultural regions confirm the effectiveness of the proposed approach. The MAVC-XAI framework achieves an accuracy of 0.938, a precision of 0.932, a recall of 0.927, an F1-score of 0.929, an mAP@50 of 0.872, and a Top-5 recognition rate of 97.8%, all significantly surpassing unimodal models such as ResNet, Swin-T, and VGGish, as well as multimodal baselines including MMBT and ViLT. Ablation experiments further validate the critical contributions of the cross-modal sampling attention and contrastive learning modules to performance enhancement. The proposed framework not only enables high-precision natural enemy identification under complex ecological conditions but also provides an interpretable and intelligent foundation for AI-driven ecological pest management and food security monitoring.

## Simple Summary

Accurate identification of natural enemies is essential for ecological pest management, yet traditional methods relying on visual inspection often fail under complex field conditions. This study proposes a multimodal recognition framework, MAVC-XAI, which integrates visual appearance and acoustic signals to improve the detection of key natural enemy species in agricultural ecosystems. The model not only achieves high recognition accuracy but also provides interpretable visualizations to reveal ecologically meaningful diagnostic features. By supporting real-time monitoring and decision-making, this approach offers a practical and intelligent tool for reducing pesticide use, enhancing biological control, and promoting sustainable agricultural production.

## 1. Introduction

Natural enemies serve as key biological control agents in agricultural ecosystems, maintaining biodiversity, reducing chemical pesticide use, and ensuring crop yield and quality [1]. Comprising insects and arthropods such as lady beetles, lacewings, parasitoid wasps, and predatory mites, they suppress pest populations through predation and parasitism, forming the foundation of sustainable pest management [2]. Under the global push for sustainable agriculture, accurate and efficient monitoring and recognition of natural enemy species and behaviors are essential for devising scientific pest control strategies, assessing ecosystem health, and predicting community dynamics [3,4]. However, recognition in real field environments remains challenging. Traditional vision-based methods, dependent on manual observation or unimodal algorithms, often degrade in open-field settings due to illumination changes, background complexity, occlusion, and low resolution [5,6]. Subtle inter-species morphological differences—such as similar color patterns among lady beetles or minute wing venation distinctions in lacewings—further complicate identification [7]. Moreover, conventional visual approaches fail to capture behavioral cues like acoustic rhythms or activity patterns, limiting ecological interpretation and dynamic monitoring capabilities [8].

With the rapid advancement of deep learning, computer vision models based on convolutional neural networks (CNNs) and vision transformers (ViT) have achieved outstanding performance in species identification and fine-grained classification tasks [9]. These methods are capable of extracting discriminative representations through multi-layer nonlinear transformations and end-to-end optimization, alleviating the limitations of handcrafted features and improving robustness [10]. Meanwhile, the progress in acoustic recognition techniques provides a complementary data modality for biodiversity monitoring [11]. By collecting field audio through high-sensitivity microphones or directional recorders and analyzing mel-spectrograms or log-spectrograms, species-specific vocalizations and temporal activity rhythms can be captured effectively [12]. Although such approaches have shown success in avian and amphibian studies, their application to small insect natural enemies remains in its infancy [13].

In recent years, multimodal deep learning has emerged as an important paradigm for biodiversity monitoring [14,15]. By integrating multiple sensing modalities such as vision and acoustics, models can establish correlations across modalities within the feature space, enabling more complete and robust species representations [16]. For example, multimodal bird identification frameworks can rely on acoustic cues to compensate for poor illumination or partial occlusion, thereby improving overall accuracy [17]. Nonetheless, existing multimodal methods remain limited in natural enemy recognition due to three primary challenges [18]. First, cross-modal alignment precision is insufficient: differences in sampling rate, temporal synchronization, and information density between vision and audio modalities hinder accurate correspondence [19]. Second, inter-species fine-grained discrimination remains weak [20]: when morphologically similar species are present, simple feature fusion fails to form distinct decision boundaries [21]. Third, ecological interpretability is lacking; most methods focus solely on classification accuracy, without providing visualized justifications for model decisions, which restricts ecological reliability and practical usability [22]. Duan et al. [23] proposed a multimodal approach combining tiny-BERT and R-CNN+ResNet-18, integrating image and textual features with linear regression and random forest ensemble learning, significantly improving pest detection accuracy and efficiency. Lyu et al. [24] developed a hybrid CNN–Transformer framework introducing a symbiotic attention mechanism and pairwise label-matching loss, achieving an F1-score of 86.5% and superior generalization in predator–pest co-occurrence detection. Cao et al. [25] designed the MCL-VD framework that fuses source code, comments, and audio spectrogram transformer (AST) modalities, employing LoRA-optimized GraphCodeBERT with multimodal contrastive learning to enhance vulnerability detection, achieving F1 improvements of 4.86–17.26% on benchmark datasets. Sun et al. [26] proposed the CCLN model integrating situation–behavior–impact(SBI) and flash EEPROM emulation (FEE) modules with dual-branch contrastive learning, leveraging weakly supervised video-level labels to align and localize audiovisual events accurately, leading to substantial performance gains in Audio-Visual Event (AVE) localization tasks.

To address the aforementioned limitations, a multimodal contrastive learning framework for natural enemy recognition and ecological interpretation (MAVC-XAI) is proposed in this study. The framework leverages the complementarity between visual and acoustic modalities, enhances fine-grained recognition through cross-species contrastive learning, and incorporates explainable artificial intelligence (XAI) to provide ecological interpretability. The main innovations are summarized as follows.

1. We constructed a multimodal natural enemy dataset consisting of synchronized visual–acoustic recordings from farmland, forest, and grassland ecosystems, covering 15 common beneficial insect species (e.g., lady beetles, lacewings, and parasitoid wasps) with detailed environmental metadata and expert annotations.
2. A multimodal Transformer fusion module was designed, employing a Swin Transformer backbone for multi-scale visual feature extraction and a CNN–Transformer hybrid for acoustic mel-spectrogram encoding. Cross-modal attention enables deep semantic alignment and fusion between the two branches.
3. A cross-species contrastive learning mechanism was established by constructing positive and negative sample pairs across species and integrating InfoNCE contrastive loss with classification loss to strengthen inter-species discriminability and reduce confusion among similar taxa.
4. A cross-modal explainability analysis module was implemented, generating Grad-CAM visual heatmaps to highlight key recognition cues such as wing veins and spot patterns, and marking acoustic spectrogram regions of model focus, thereby providing interpretable ecological evidence through multimodal visualization.

In the field of agricultural economics, intelligent recognition and ecological monitoring of natural enemies possess not only biological control significance but also direct implications for agricultural productivity and economic returns. With the advancement of green pest management and sustainable agriculture, natural enemies serve as critical ecological regulators, whose identification and dynamic monitoring provide essential data support for pesticide reduction and yield improvement. By constructing multimodal recognition models, intelligent analyses of population distribution, activity cycles, and ecological interactions of natural enemies can be achieved, thereby offering data-driven decision-making guidance for agricultural producers. For instance, in the fruit and vegetable industry, fluctuations in the abundance and activity levels of natural enemies can serve as key economic indicators for predicting pest outbreaks and assessing ecological risks. At the regional management level, intelligent recognition systems can assist in building coupled “control–yield–profit” models, enabling precise pesticide application and optimizing input–output efficiency. Furthermore, the visual–acoustic fusion framework significantly reduces manual inspection costs and enhances the digitalization level of farmland management, facilitating the transformation of agricultural production from experience-based to data-driven paradigms. This technological pathway not only contributes to ensuring food security and ecological stability but also injects new intelligent growth momentum into the modern agricultural economic system.

## 2. Related Work

### 2.1. Natural Enemy Recognition and Multimodal Feature Fusion

In the field of natural enemy recognition, traditional computer vision approaches primarily rely on image classification, object detection, and instance segmentation techniques, performing species discrimination through the extraction of morphological features [27]. The core principle of these methods is to map an input image into a high-dimensional feature space, where classification or similarity-based metrics are applied for category prediction [28]. For natural enemy identification, single-modality vision-based methods can achieve high accuracy under standardized conditions; however, their performance deteriorates significantly in outdoor environments due to illumination variation, occlusion, and background complexity, which affect the stability of mapping [29]. Recently, multimodal feature fusion has emerged as an effective strategy to enhance model robustness [30]. Particularly in biological identification tasks involving birds or insects, the combination of visual and acoustic features provides complementary cues [31]. The fundamental concept involves extracting modality-specific feature vectors, which are then fused through a joint function. Common fusion strategies include feature concatenation, weighted summation, and nonlinear fusion based on attention mechanisms [32]. Nevertheless, in natural enemy recognition, discrepancies in temporal and spatial sampling frequencies between visual and acoustic signals often result in poor cross-modal alignment accuracy [33]. If the sampling timestamp sets of the two modalities are represented as $T_{v}$ and $T_{a}$, a temporal mapping function $g:T_{v}\to T_{a}$ must be constructed to minimize the inter-modal distance:

(1) $$\min_{g}\;{\left|f_{v}\left(x_{v}\left(t\right)\right)-f_{a}\left(x_{a}\left(g\left(t\right)\right)\right)\right|}_{2}^{2},$$

where $f_{v}$ and $f_{a}$ denote the visual and acoustic feature extractors, and $x_{v}\left(t\right)$ and $x_{a}\left(t\right)$ represent modality data at time *t*. Under limited data conditions, learning such mappings accurately becomes challenging, posing a major bottleneck for current multimodal natural enemy recognition methods.

### 2.2. Applications of Contrastive Learning in Fine-Grained Biological Recognition

Contrastive learning has demonstrated strong discriminative capability in fine-grained biological recognition by constructing positive and negative sample pairs to minimize intra-class distances while maximizing inter-class separations [34]. Given a sample $x_{i}$ with a positive pair $x_{i}^{+}$ and a set of negative samples $x_{j}^{-}$, the typical InfoNCE loss is defined as:

(2) $$L_{\mathrm{InfoNCE}}=-\log\frac{\exp(\mathrm{sim}\left(z_{i},z_{i}^{+}\right)/\tau )}{\exp\left(\mathrm{sim}\left(z_{i},z_{i}^{+}\right)/\tau \right)+\sum_{j}\exp\left(\mathrm{sim}\left(z_{i},z_{j}^{-}\right)/\tau \right)},$$

where $z_{i}=f_{\theta }\left(x_{i}\right)$ denotes the embedding of sample $x_{i}$, sim(·,·) represents the similarity measure (e.g., cosine similarity), and τ is the temperature parameter. In fine-grained recognition, this mechanism compels the model to capture subtle morphological distinctions, allowing it to establish clearer decision boundaries among morphologically similar species [35]. Instance-level contrastive learning focuses on ensuring consistency across different viewpoints or modalities of the same individual, whereas class-level contrastive learning aggregates embeddings of the same class around a class centroid vector $c_{y}$, enhancing intra-class compactness by minimizing $|z_{i}-c_{y_{i}}{|}_{2}^{2}$. For natural enemy identification, incorporating contrastive learning within a multimodal framework not only alleviates confusion among similar species but also improves feature generalization under limited data conditions [36].

### 2.3. Explainable Artificial Intelligence in Ecological Monitoring

In the domain of explainable artificial intelligence (XAI), techniques such as Grad-CAM and Score-CAM have provided intuitive tools for understanding the decision-making processes of deep neural networks [37]. Taking Grad-CAM as an example, the method computes channel-wise weights $\alpha_{k}^{c}$ for the feature maps $A^{k}$ in the final convolutional layer with respect to the predicted score $y^{c}$ of class *c*:

(3) $$\alpha_{k}^{c}=\frac{1}{Z}\sum_{i}\sum_{j}\frac{\partial y^{c}}{\partial A_{ij}^{k}},$$

where *Z* is a normalization constant and i,j indicate spatial positions. The class activation map is then generated as:

(4) $$L_{\mathrm{Grad}-\mathrm{CAM}}^{c}=\mathrm{ReLU}\left(\sum_{k}\alpha_{k}^{c}A^{k}\right).$$

This heatmap highlights the regions of the input image that contribute most to the classification outcome. In the acoustic modality, mel-spectrograms can be treated analogously to images, enabling the same gradient-based weighting strategy to locate important regions in the time–frequency domain [38]. However, existing ecological monitoring studies are predominantly restricted to single-modality interpretation, and in the context of insect natural enemy recognition, no systematic dual-modality (visual and acoustic) interpretability framework has been established, thereby limiting traceability and credibility in ecological applications [39]. By normalizing and fusing visual and acoustic interpretability results $L_{v}$ and $L_{a}$ through a weighted summation,

(5) $$L_{\mathrm{fusion}}=\beta L_{v}+\left(1-\beta \right)L_{a},$$

where β∈[0,1] denotes the modality weight coefficient, a more comprehensive interpretive basis can be provided for ecologists, enabling joint analysis of behavioral patterns and morphological traits.

### 2.4. Application of Intelligent Natural Enemy Recognition in Agricultural Economics

The application of intelligent natural enemy recognition technology has become a critical component in promoting sustainable agricultural development within the field of agricultural economics. By integrating artificial intelligence with ecological monitoring, real-time assessments of natural enemy population dynamics, pest control effectiveness, and farmland ecological balance can be achieved, thereby providing data-driven decision support for agricultural production. For instance, in the cultivation of high-value economic crops, multimodal recognition-based monitoring systems can be employed to evaluate the input–output ratio of pest management strategies, optimizing resource allocation and reducing pesticide costs. Moreover, this technology can be combined with economic modeling to predict the impact of pest outbreaks on yield and revenue, offering evidence-based guidance for agricultural management and policy formulation. The incorporation of intelligent natural enemy recognition transforms ecological pest control from a purely biological issue into a key technological pathway for enhancing efficiency and profitability within modern agricultural economic systems.

## 3. Materials and Method

### 3.1. Data Collection

**Figure 1:**
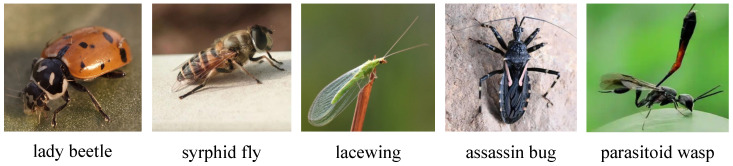
Dataset samples.

Data collection for this study was conducted from March 2023 to October 2024 across representative agricultural ecosystems located in Bayan Nur (Inner Mongolia), Tangshan (Hebei), Mengzi (Yunnan) and the Internet, encompassing farmland, forestland, and grassland habitats, as shown in Table 1. To comprehensively capture multimodal characteristics of natural enemies under natural conditions, synchronized acquisition systems were deployed at each site, consisting of a high-definition RGB camera (resolution 3840 × 2160, frame rate 30 fps) and a directional condenser microphone (frequency response 20 Hz–20 kHz, sampling rate 44.1 kHz). Both devices were calibrated through a unified time synchronization module to ensure temporal alignment between visual and acoustic streams. The acquisition setup was mounted on an adjustable tripod approximately 1.2 m above ground, with the camera angle dynamically adjusted according to vegetation height and lighting conditions. Each site was recorded for 3 h daily, divided into morning (6:00–9:00), midday (12:00–15:00), and evening (17:00–20:00) sessions, thereby encompassing the major activity periods of natural enemies. The visual data primarily documented the behavioral dynamics of individual predators on crop leaves, stems, and soil surfaces, covering a broad range of illumination intensities, background complexities, and occlusion levels to enhance environmental diversity. Approximately 32,000 images were collected, including around 8000 of lady beetles, 6500 of lacewings, and 5400 of parasitoid wasps, in addition to spiders, assassin bugs, and syrphid flies, as shown in Figure 1. Each image was manually annotated with species labels, bounding boxes, and pose information.

Acoustic recordings were captured synchronously with visual acquisition. Each microphone operated in directional mode and was positioned approximately 20 cm from the camera to maximize the capture of vocal signals corresponding to individuals in the visual frame. The raw audio recordings were transformed into Mel-spectrograms via time–frequency analysis and manually cross-validated to ensure precise correspondence between audio and visual samples. Specifically, two trained entomology experts independently inspected a stratified subset of synchronized clips, verifying that the focal individual in the visual frame corresponded to the dominant acoustic source in the spectrogram. Samples with ambiguous correspondence, such as those containing overlapping calls from multiple individuals or strong off-frame sound sources, were either discarded or labeled as weakly aligned and excluded from supervised multimodal training. In scenes where multiple insects were present, only clips in which a single focal natural enemy occupied the central region of the frame and dominated the acoustic energy were retained as fully aligned pairs. In total, 18,500 acoustic clips were obtained, of which approximately 62% contained valid sound segments. During data collection, environmental parameters including temperature, humidity, and light intensity were simultaneously recorded using DHT22 and LDR sensors at a frequency of one sample per minute. These environmental records were used for subsequent behavioral analysis and ecological variable correction in modeling. After temporal alignment, all data were integrated into unified sample pairs, resulting in the first multimodal natural enemy dataset combining visual, acoustic, and environmental information. This dataset provides a comprehensive foundation for multimodal feature learning and ecologically interpretable recognition.

### 3.2. Data Preprocessing and Augmentation

In multimodal natural enemy recognition tasks, data preprocessing and augmentation play a crucial role in improving model generalization and robustness. Since field-collected images and acoustic data are often affected by illumination variations, occlusion, and environmental noise, directly training models with raw data may cause the feature extractor to learn environment-sensitive patterns, thereby reducing recognition performance under real-world conditions. Consequently, it is necessary to apply a series of augmentation operations that preserve essential biological characteristics while enabling the model to learn more stable and discriminative multimodal representations.

For the visual modality, common data augmentation methods include random cropping, brightness and contrast adjustment, and background replacement. The principle of random cropping is to select a random spatial region of the original image I(x,y) for scaling or padding, thereby altering the target’s scale and spatial distribution. Assuming a cropping ratio α∈(0,1], the cropped image can be expressed as

(6) $$I_{\mathrm{crop}}\left(x^{\prime },y^{\prime }\right)=I\left(\alpha x+\Delta_{x},\alpha y+\Delta_{y}\right),$$

where $\Delta_{x}$ and $\Delta_{y}$ denote random translation offsets. Brightness and contrast adjustment modifies the pixel intensity distribution through a linear transformation. Given a pixel value *p*, a brightness bias *b*, and a contrast coefficient *c*, the enhanced pixel value is defined as

(7) $$p^{\prime }=\mathrm{clip}\left(c\cdot p+b,\;0,\;255\right),$$

where the clip operation ensures that results remain within valid pixel ranges. Background replacement relies on a target detection or segmentation model to generate a foreground mask M(x,y)∈{0,1}, while the background region is replaced by a randomly selected environmental scene B(x,y), producing a new image as

(8) $$I_{\mathrm{bg}}\left(x,y\right)=M\left(x,y\right)\cdot I\left(x,y\right)+\left(1-M\left(x,y\right)\right)\cdot B\left(x,y\right).$$

This process significantly enhances the model’s generalization capability across different ecological environments.

For the acoustic modality, data augmentation aims to improve robustness to environmental noise, temporal shifts, and spectral distortions. The time-shifting operation translates the acoustic signal s(t) along the temporal axis, which can be formulated as

(9) $$s_{\mathrm{shift}}\left(t\right)=s\left(t+\delta_{t}\right),$$

where $\delta_{t}$ represents the random temporal offset. Noise addition superimposes random noise n(t) on the original signal, typically modeled as $n\left(t\right)∼N\left(0,\sigma^{2}\right)$. The enhanced signal is thus given by

(10) $$s_{\mathrm{noise}}\left(t\right)=s\left(t\right)+\lambda n\left(t\right),$$

where λ controls the noise intensity. Spectral smoothing is performed on the Mel-spectrogram representation S(f,τ), where *f* denotes frequency and τ represents the time frame index. Convolution kernels $K_{f}$ and $K_{\tau }$ are applied along frequency and time axes, respectively:

(11) $$S_{\mathrm{smooth}}\left(f,\tau \right)=\left(S*K_{f}*K_{\tau }\right)\left(f,\tau \right).$$

This operation suppresses high-frequency noise interference and emphasizes the main structural patterns of acoustic features.

Multimodal alignment serves as a critical step in this study, aiming to pair visual frames with corresponding acoustic segments collected within the same time window to form effective multimodal training samples. Let the visual sampling timestamps be $T_{v}=\left\{t_{v}^{1},t_{v}^{2},\ldots ,t_{v}^{N_{v}}\right\}$ and the acoustic timestamps be $T_{a}=\left\{t_{a}^{1},t_{a}^{2},\ldots ,t_{a}^{N_{a}}\right\}$. The goal is to find a mapping function $g:T_{v}\to T_{a}$ such that visual frames $x_{v}\left(t_{v}\right)$ are temporally aligned with their corresponding acoustic segments $x_{a}\left(g\left(t_{v}\right)\right)$. The optimization objective for temporal alignment is defined as

(12) $$\min_{g}\;\frac{1}{N_{v}}\sum_{i=1}^{N_{v}}\left|t_{v}^{i}-g\left(t_{v}^{i}\right)\right|.$$

In practical implementations, *g* is typically obtained through nearest-neighbor matching or dynamic time warping (DTW). After alignment, each paired sample is represented as $\left(x_{v}^{i},x_{a}^{i}\right)$ and jointly fed into the multimodal network for feature learning during training.

It is noteworthy that a strong interdependence exists between data augmentation and multimodal alignment. During augmentation, it is essential to ensure that the temporal consistency between visual and acoustic modalities remains intact. For a paired sample $\left(x_{v}\left(t\right),x_{a}\left(t\right)\right)$ at timestamp *t*, if a time shift $\delta_{t}$ is introduced in the acoustic modality, an equivalent delay or corresponding sample adjustment must be applied to the visual modality to maintain semantic consistency across modalities. This consistency constraint can be expressed as

(13) $$\forall t,\;|f_{v}\left(x_{v}\left(t\right)\right)-f_{a}\left(x_{a}\left(t+\delta_{t}\right)\right){|}_{2}\leq \epsilon ,$$

where $f_{v}$ and $f_{a}$ denote the visual and acoustic feature extraction functions, respectively, and ϵ represents the allowable cross-modal deviation threshold.

### 3.3. Proposed Method

The overall workflow of the proposed method is as follows. After temporal synchronization, image frames and their corresponding acoustic segments are respectively fed into a dual-architecture spatiotemporal feature extraction network. In this network, the visual branch extracts local textures through convolutional operations and then models global dependencies using a Transformer, whereas the acoustic branch takes Mel-spectrograms as input, capturing band energy patterns via convolution and learning long-term temporal dependencies through a Transformer. Both branches produce visual and acoustic embeddings with unified dimensionality. These embeddings are subsequently processed by a cross-modal sampling attention mechanism, in which visual and acoustic embeddings act as mutual queries and key–value pairs for bidirectional interaction. Noise-adaptive sampling weights are introduced to generate aligned and complementary fused representations. The fused representation is then used in two parallel paths: one passes through a classification head to produce species predictions, and the other enters a cross-species contrastive learning module, where positive samples from the same species and hard negatives from morphologically similar species are used to construct contrastive objectives. This process enforces intra-class compactness and inter-class separation, thereby strengthening fine-grained decision boundaries. During training, the model is jointly optimized by minimizing the weighted sum of classification and contrastive losses, whereas during inference, the final class probabilities are directly output. To ensure ecological interpretability, intermediate features and attention weights from the classification branch are fed into an interpretability generation module, which produces visual saliency maps and acoustic time–frequency attention maps. These two modalities are further fused to generate a joint heatmap that highlights key ecological cues, which is output together with the classification result for expert interpretation.

#### 3.3.1. Dual-Architecture Spatiotemporal Feature Extraction Network

**Figure 2:**
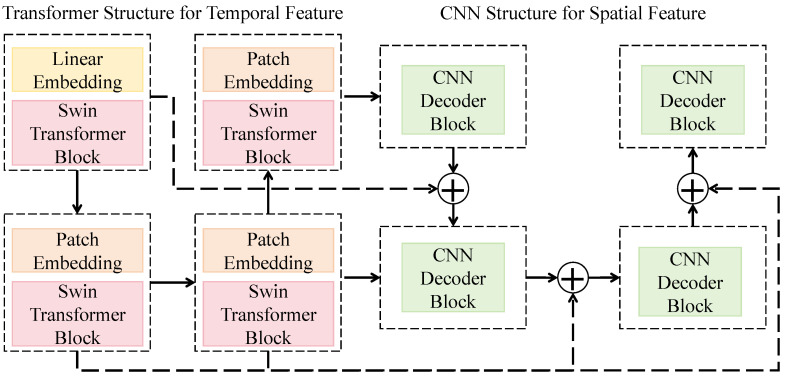
Schematic of the Dual-Architecture Spatiotemporal Feature Extraction Network.

The dual-architecture spatiotemporal feature extraction network is designed to simultaneously capture the spatial visual features and temporal acoustic features of natural enemies, enabling joint representation of morphological and behavioral signals. As shown in Figure 2, the overall structure adopts a parallel design consisting of visual and acoustic branches that share the same feature dimensionality and encoding rules to maintain modality alignment. The visual branch is based on a hybrid architecture combining an improved Swin Transformer with a CNN. The input image first passes through a linear embedding module that projects raw pixels into a high-dimensional feature space to form initial patch embeddings. It is then processed by multiple Swin Transformer blocks, each containing local window attention and shifted window mechanisms for capturing both local structural information and global spatial dependencies. Multi-stage features are hierarchically downsampled to form multi-scale representations, and a convolutional decoder is applied at the end to reconstruct spatial distributions, thereby generating visual embeddings that incorporate both global contextual awareness and local detail sensitivity.

The acoustic branch takes the Mel-spectrogram as input and employs an identical-sized patch embedding module to generate time–frequency patch vectors. These are fed into a Transformer to model temporal dependencies and frequency pattern variations. Each Transformer layer applies multi-head self-attention to compute inter-frame correlations, while residual connections and layer normalization ensure gradient stability. The Swin Transformer stages use channel dimensions of 96, 192, 384, and 768, with each block containing two multi-head attention modules using 3, 6, 12, and 24 heads, respectively. The CNN decoder consists of four convolutional blocks with 3 × 3 kernels, a stride of 2, and the GELU activation function, which balances nonlinear expressiveness and numerical stability. The acoustic Transformer adopts the same depth and channel configuration as the visual branch but incorporates temporal positional encoding to enhance rhythm-aware event perception. Mathematically, the network can be expressed as $F_{v}=\Phi_{CNN+Swin}\left(I_{v}\right)$ and $F_{a}=\Phi_{Trans}\left(S_{a}\right)$, where $F_{v}$ and $F_{a}$ denote the extracted visual and acoustic feature mappings, and $I_{v}$ and $S_{a}$ represent the respective input modalities. A shared projection matrix $W_{p}\in R^{d_{in}\times d}$ with a unified feature dimension *d* is applied to align the feature spaces, allowing the subsequent cross-modal attention fusion to operate directly within these aligned subspaces.

This architecture effectively combines spatial detail extraction and temporal dynamics modeling, thereby enhancing joint recognition performance of visual morphological traits and acoustic behavioral features under complex ecological conditions. The hierarchical window attention of the Transformer ensures efficient global modeling, while the convolutional decoder restores spatial resolution and strengthens local texture perception. Consequently, the model maintains strong discriminative capability and generalization performance even in environments characterized by morphological similarity and high noise interference.

#### 3.3.2. Cross-Modal Sampling Attention Mechanism

**Figure 3:**
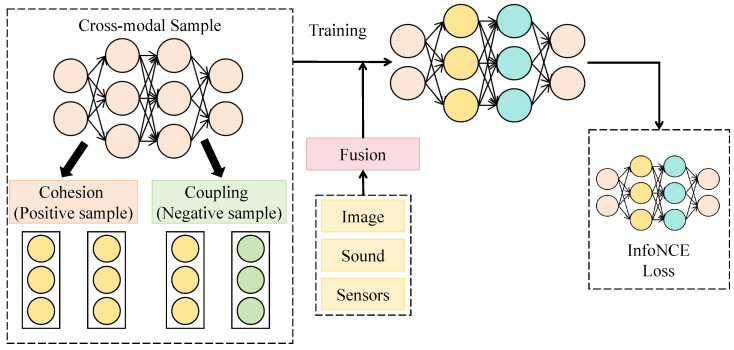
Illustration of the Cross-Modal Sampling Attention Mechanism.

The cross-modal sampling attention mechanism is designed to establish a dynamic and bidirectional interaction between visual and acoustic embeddings, thereby achieving deep alignment and complementary representation across modalities. Unlike traditional self-attention mechanisms that compute feature dependencies within a single modality, the cross-modal attention mechanism performs interactive sampling between visual and acoustic features, allowing both modalities to guide and refine each other’s semantic distributions and form a consistent joint representation across heterogeneous data. As shown in Figure 3, this module takes visual embeddings $V\in R^{H_{v}\times W_{v}\times C_{v}}$ and acoustic embeddings $A\in R^{H_{a}\times W_{a}\times C_{a}}$ as input. After linear projection to a unified dimensionality d=512, they are fed into four layers of bidirectional interaction blocks. Each layer comprises query–key mapping, attention computation, and fusion normalization, with channel dimensions of 128, 256, 512, and 512, corresponding to spatial scales of 32×32, 16×16, 8×8, and 4×4, respectively. Within each layer, the visual features act as queries while the acoustic features serve as keys and values to compute cross-modal attention weights; the roles are then reversed to enable bidirectional semantic complementarity.

The core computation of this mechanism is defined as:

(14) $$\alpha_{va}=\mathrm{Softmax}\left(\frac{\left(VW_{q}\right){\left(AW_{k}\right)}^{T}}{\sqrt{d}}\right),\;\alpha_{av}=\mathrm{Softmax}\left(\frac{\left(AW_{q}\right){\left(VW_{k}\right)}^{T}}{\sqrt{d}}\right),$$

where $W_{q},W_{k}\in R^{d\times d}$ are learnable matrices. Based on these weights, the fused features are updated as:

(15) $$V^{\prime }=\alpha_{va}\left(AW_{v}\right),\;A^{\prime }=\alpha_{av}\left(VW_{v}\right),$$

followed by residual connections and layer normalization for stable outputs. Mathematically, the cross-modal sampling mechanism can be interpreted as finding an optimal matching function $f^{*}\left(V,A\right)$ on the manifolds of the two modalities, with the objective of minimizing the inter-modal discrepancy $d(V^{\prime },A^{\prime })$ to achieve alignment in the feature space. Compared with single-modal self-attention, this mechanism theoretically extends the original attention kernel into a cross-modal kernel function with enhanced nonlinear mapping capacity, effectively reducing semantic drift between modalities.

The cross-modal sampling attention mechanism is jointly applied with the subsequent cross-species contrastive learning module. Through contrastive loss optimization in the fused feature space, the mechanism further compresses intra-class cross-modal discrepancies while enlarging inter-class distances, thereby forming a stable multimodal decision boundary. Mathematically, when the InfoNCE loss term $L_{con}$ is optimized on top of the mechanism’s outputs, it is equivalent to introducing a cross-modal constraint term $\min|V^{\prime }-A^{\prime }{|}_{2}$ in the embedding space, ensuring not only classification-level consistency but also representational alignment. This design enables the model to achieve semantic coherence and complementary fusion across modalities in complex ecological environments, significantly enhancing the stability and robustness of natural enemy recognition and behavioral discrimination.

#### 3.3.3. Cross-Species Contrastive Learning Module

**Figure 4:**
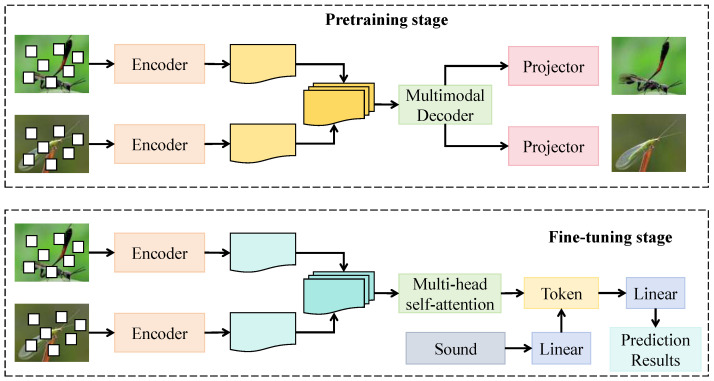
Schematic of the Cross-Species Contrastive Learning Module.

The cross-species contrastive learning module is designed to construct a multimodal joint representation space that enhances the discriminative ability of the model between morphologically similar species while maintaining semantic consistency across modalities. As shown in Figure 4, this module consists of dual encoders, a projection head, and a multimodal decoder, corresponding to the pretraining and fine-tuning stages. During pretraining, the visual and acoustic encoders operate in parallel, where the visual encoder receives $F_{v}\in R^{H_{v}\times W_{v}\times C_{v}}$ ($H_{v}=8,W_{v}=8,C_{v}=512$) from the preceding module, and the acoustic encoder processes $F_{a}\in R^{H_{a}\times W_{a}\times C_{a}}$ ($H_{a}=8,W_{a}=4,C_{a}=512$). Both encoders undergo three residual attention layers before passing into a shared multimodal projection head, which maps the features into a unified embedding dimension d=256. The projection head consists of two linear layers, with the first layer having a width of 512 and employing the GELU activation function, while the second layer outputs 256-dimensional vectors normalized by L2 regularization to constrain representation distributions. In the fine-tuning phase, a multi-head self-attention structure with eight heads and an embedding dimension of 256 is introduced, where each attention head learns semantic alignment relationships among different species. The fused features are subsequently decoded and fed into the classification layer to achieve cross-species feature alignment and category enhancement.

Mathematically, let $z_{v},z_{a}\in R^{d}$ denote the projected visual and acoustic embeddings, respectively, and define their joint representation as $z_{m}=\phi \left(z_{v},z_{a}\right)$, where ϕ(·) denotes the multimodal decoding function. To achieve cross-species contrastive constraints, a dynamic pairing strategy is introduced by computing the similarity matrix $S\in R^{N\times N}$ among species embeddings to select positive and negative pairs. Positive samples correspond to different individuals from the same species, while negative samples originate from morphologically or acoustically similar but distinct species. The optimization objective minimizes the mutual information loss between cross-modal embeddings, defined as:

(16) $$L_{cs}=\frac{1}{N}\sum_{i=1}^{N}\left[\log\frac{\exp(\psi \left(z_{m,i},z_{m,i}^{+}\right)/\tau )}{\sum_{j=1}^{N}\exp\left(\psi \left(z_{m,i},z_{m,j}^{-}\right)/\tau \right)}\right],$$

where ψ(·) denotes the multimodal similarity function and τ represents the temperature parameter. To prevent embedding collapse and enhance cross-species separation, a margin constraint term is introduced:

(17) $$L_{margin}=\max(0,\delta -|z_{m,i}-z_{m,i}^{+}{|}_{2}^{2}+|z_{m,i}-z_{m,j}^{-}{|}_{2}^{2}),$$

and the overall loss function is defined as:

(18) $$L_{total}=L_{cls}+\lambda_{1}L_{cs}+\lambda_{2}L_{margin},$$

where $\lambda_{1}$ and $\lambda_{2}$ control the relative weights of cross-modal consistency and cross-species discrimination, respectively.

Theoretically, when $\nabla_{z_{m}}L_{total}=0$, the model reaches an optimal state of cross-modal embedding alignment. Proof is as follows: let $z_{m}^{*}$ denote the optimal embedding. When both the contrastive and margin losses converge to their minima, it holds that $|z_{m,i}-z_{m,i}^{+}{|}_{2}<{\left|z_{m,i}-z_{m,j}^{-}\right|}_{2}$. In this case, the gradient direction is determined by an attractive term for positive samples and a repulsive term for negative samples, whose directional derivative is negative. This guarantees that during convergence, the embeddings maintain intra-class compactness and inter-class dispersion, achieving stable cross-species separation.

The module is jointly employed with the preceding cross-modal sampling attention mechanism, where the latter provides temporally and spatially fused embeddings and the former reshapes discriminative boundaries through contrastive optimization. This end-to-end integration yields a coherent multimodal feature flow. By introducing a cross-species metric space and semantic margin constraints, the model can reliably identify morphologically similar but behaviorally distinct species in complex ecological environments, thereby enhancing generalization ability and ecological interpretability while providing a robust foundation for ecological recognition tasks.

#### 3.3.4. Explainable Generation Module

The explainable generation module is attached in parallel after the classification heads of the visual and acoustic branches, employing lightweight interpreter heads to transform intermediate feature maps and attention tensors into cross-modal readable saliency representations. In the visual interpreter, the feature tensor with dimensions 8×8×512 is extracted from the final convolutional layer of the visual branch. A 1×1 convolution is first applied to reduce the channel dimension to 128, followed by a deconvolution sequence k=4,s=2,c:128→64→k=4,s=2,c:64→32→k=4,s=2,c:32→1, which progressively upsamples the tensor to 64×64×1, forming the visual heatmap $H_{v}^{c}$. The acoustic interpreter reshapes the final-layer Transformer tokens of the acoustic branch into a 8×4×512 tensor, compresses channels to 128 via a 1×1 convolution, and performs an equivalent deconvolution sequence to upsample the representation to 64×64×1, generating the acoustic heatmap $H_{a}^{c}$.

To avoid redundancy with earlier formulations, the visual branch employs the Grad-CAM++ weighting formulation. Denoting convolutional features as $A^{k}$ and the class logit as $y^{c}$, the weight is defined as

(19) $$\alpha_{k}^{c}=\frac{\sum_{i,j}\frac{\partial^{2}y^{c}}{\partial {\left(A_{ij}^{k}\right)}^{2}}}{2\sum_{i,j}\frac{\partial^{2}y^{c}}{\partial {\left(A_{ij}^{k}\right)}^{2}}+\sum_{i,j}A_{ij}^{k}\frac{\partial^{3}y^{c}}{\partial {\left(A_{ij}^{k}\right)}^{3}}},$$

and the spatial saliency map is generated as $H_{v}^{c}=\mathrm{ReLU}\;\left(\sum_{k}\alpha_{k}^{c}A^{k}\right)$. The acoustic branch adopts integrated gradients between the Mel-spectrogram *S* and a baseline $S^{\prime }$, accumulating attribution as

(20) $$H_{a}^{c}=\left(S-S^{\prime }\right)⊙\int_{0}^{1}\frac{\partial y^{c}\;\left(S^{\prime }+t\left(S-S^{\prime }\right)\right)}{\partial S}\;dt,$$

which is then normalized and aligned to a unified resolution. The two saliency maps are fused by a gating operator G consisting of two 1×1 convolutional layers (2→16→1) with an intermediate GELU activation, producing a pixel-wise gating map $G=\sigma (G\left(\left[H_{v}^{c},H_{a}^{c}\right]\right))$. The final cross-modal explanation map is then formulated as

(21) $$H^{c}=G⊙H_{v}^{c}+\left(1-G\right)⊙H_{a}^{c},$$

where σ denotes the Sigmoid function and $H^{c}\in R^{64\times 64}$. To enhance smoothness and readability, an isotropic total variation regularization term $|\nabla H^{c}{|}_{1}$ is appended as a consistency constraint during training.

This module is jointly optimized with the cross-modal sampling attention mechanism through a distribution alignment regularizer. Denoting the marginal spatial distribution of cross-modal attention as $M^{c}$, both $H^{c}$ and $M^{c}$ are normalized using the Softmax operation to obtain ${\tilde{H}}^{c}$ and ${\tilde{M}}^{c}$. The explanation–attention alignment term is then defined as

(22) $$L_{\mathrm{align}}=D_{\mathrm{KL}}\;\left({\tilde{H}}^{c}\;∥\;{\tilde{M}}^{c}\right),$$

and the total explanation loss is expressed as $L_{\mathrm{xai}}=\mu_{1}{\left|\nabla H^{c}\right|}_{1}+\mu_{2}L_{\mathrm{align}}$. Theoretical analysis shows that if G∈[0,1] and $H_{v}^{c},H_{a}^{c}\;\geq \;0$, then for any non-negative weighting function *w*,

(23) $$\int wH^{c}=\int w\left(GH_{v}^{c}+\left(1-G\right)H_{a}^{c}\right)\in \left[\min\left(\int wH_{v}^{c},\int wH_{a}^{c}\right),\;\max\left(\cdot \right)\right],$$

indicating that the fused explanation’s contribution in any region is no less than the lower bound of the weaker modality, thus improving saliency where information is complementary. Moreover, since $D_{\mathrm{KL}}\left({\tilde{H}}^{c}∥{\tilde{M}}^{c}\right)\geq 0$ and reaches zero if and only if ${\tilde{H}}^{c}={\tilde{M}}^{c}$, minimizing $L_{\mathrm{align}}$ aligns the explanation distribution with the marginal attention distribution, yielding a stable fixed point consistent with feature interaction. Such consistency enhances cross-scene robustness of discriminative regions. When combined with the cross-species contrastive learning framework, an additional class mask $\Pi_{c}$ is applied to gate the feature activations *F* guided by $H^{c}$, minimizing

(24) $$R_{\mathrm{gate}}=|F-\Pi_{c}⊙F{|}_{2}^{2},$$

which concentrates intra-class compactness within high-response explanatory regions, thereby reinforcing separability among fine-grained neighboring classes and reducing overfitting induced by spurious background correlations, as illustrated in Algorithm 1.
**Algorithm 1:** Explainable Analysis Module (EAM) Forward and Training Pseudocode**Input**: Visual feature map $A\in R^{8\times 8\times 512}$, raw audio *x*, class label *c*, cross-modal attention marginal $M^{c}$, class gating mask $\Pi_{c}$**Output**: Cross-modal heatmap $H^{c}$, explanation loss $L_{\mathrm{xai}}$**Visual explanation branch:**$A_{v}\leftarrow {\mathrm{Conv}}_{1\times 1}\left(A;512\to 128\right)$//(Channel reduction($H_{v}^{↑}\leftarrow {\mathrm{Deconv}}_{k=4,s=2}^{\times 3}\left(A_{v};128\to 64\to 32\to 1\right)$//(Upsampling 8×8→64×64($H_{v}\leftarrow {\mathrm{GradCAM}}^{++}\left(A,c\right)$//(Generate visual saliency map($H_{v}\leftarrow \mathrm{ResizeNorm}\left(H_{v},64\times 64\right)$//(Align spatial size and normalize(**Acoustic explanation branch:**S←MelSpec(x)//(Convert audio to Mel-spectrogram(T←Tok2Grid(S)//(Reshape to 8×4×512 grid($A_{a}\leftarrow {\mathrm{Conv}}_{1\times 1}\left(T;512\to 128\right)$//(Channel reduction($H_{a}^{↑}\leftarrow {\mathrm{Deconv}}_{k=4,s=2}^{\times 3}\left(A_{a};128\to 64\to 32\to 1\right)$//(Upsampling 8×4→64×64($H_{a}\leftarrow \mathrm{IntegratedGradients}\left(S,c\right)$//(Generate time–frequency saliency map($H_{a}\leftarrow \mathrm{ResizeNorm}\left(H_{a},64\times 64\right)$**Gated fusion:**$U\leftarrow [H_{v},H_{a}]$//(Concatenate along channel dimension (64×64×2)($G\leftarrow \sigma \;\left({\mathrm{Conv}}_{1\times 1}\left(\mathrm{GELU}({\mathrm{Conv}}_{1\times 1}\left(U;2\to 16\right));16\to 1\right)\right)$//(Pixel-wise gating mask G∈[0,1]($H^{c}\leftarrow G⊙H_{v}+\left(1-G\right)⊙H_{a}$//(Cross-modal fused explanation map(**Training-phase losses:**${\tilde{H}}^{c}\leftarrow \mathrm{Softmax}\left(\mathrm{vec}\left(H^{c}\right)\right)$, ${\tilde{M}}^{c}\leftarrow \mathrm{Softmax}\left(\mathrm{vec}\left(M^{c}\right)\right)$//(Distribution normalization($L_{\mathrm{align}}\leftarrow D_{\mathrm{KL}}\left({\tilde{H}}^{c}∥{\tilde{M}}^{c}\right)$//(Attention–explanation alignment loss($L_{\mathrm{tv}}\leftarrow {∥\nabla H^{c}∥}_{1}$//(Spatial smoothness regularization(F←BackboneFeat(·), $R_{\mathrm{gate}}\leftarrow {∥F-\Pi_{c}⊙F∥}_{2}^{2}$//(Class-wise gating regularization($L_{\mathrm{xai}}\leftarrow \mu_{1}L_{\mathrm{tv}}+\mu_{2}L_{\mathrm{align}}+\mu_{3}R_{\mathrm{gate}}$//(Total explanation loss(**return**$H^{c},\;L_{\mathrm{xai}}$

### 3.4. Experimental Environment

#### 3.4.1. Hardware and Software

The experimental setup was deployed on a high-performance computing server equipped with an Intel Xeon Gold series multi-core processor operating at a base frequency of 2.90 GHz, providing substantial parallel computational capacity. A total of 256 GB of memory was configured to support efficient data loading and intermediate feature caching during large-scale multimodal training. The system utilized an NVIDIA A100 GPU with 80 GB of video memory, ensuring sufficient capacity for the training of deep Transformer and convolutional networks under large batch sizes while significantly reducing model iteration time. In addition, high-speed NVMe solid-state drives were employed to enhance data read/write performance and mitigate potential I/O bottlenecks that could affect training efficiency.

#### 3.4.2. Hyperparameters

During experimentation, the dataset was divided into training, validation, and testing subsets with proportions of 70%, 15%, and 15%, respectively. This partitioning ensured sufficient data for model optimization while maintaining independent validation and testing subsets for unbiased generalization monitoring and final performance evaluation. The batch size was set to B=32, and the initial learning rate η was defined as $1\times 10^{-4}$. The AdamW optimizer was employed, with a weight decay coefficient λ=0.05 to mitigate overfitting, and default momentum parameters $\beta_{1}=0.9$ and $\beta_{2}=0.999$. The total number of training epochs was E=100, and an early stopping criterion was applied based on validation performance. A cosine annealing learning rate scheduler was utilized, defined as

(25) $$\eta_{t}=\eta_{\min}+\frac{1}{2}\left(\eta_{\max}-\eta_{\min}\right)\left(1+\cos\left(\frac{t}{T}\pi \right)\right),$$

where *t* denotes the current iteration step and *T* represents the total number of iterations, enabling gradual reduction of the learning rate to facilitate convergence during later training stages. Additionally, a k=5 fold cross-validation strategy was adopted, in which the dataset was partitioned into *k* non-overlapping subsets of approximately equal size. In each iteration, k−1 subsets were used for training and the remaining subset for validation. This process was repeated *k* times, and the results were averaged to minimize performance fluctuations caused by random data partitioning and to enhance the robustness of the experimental conclusions.

#### 3.4.3. Baseline Models

In this study we adopt six baseline models in order to cover unimodal vision, unimodal acoustics, and recent multimodal Transformers. For the visual modality we use ResNet [40] and Swin-T [41]. These two networks represent the mainstream convolutional and Transformer based backbones and are widely used in image recognition, which makes them suitable references for our visual branch. For the acoustic modality we select VGGish [42], a standard Mel-spectrogram encoder that has been extensively applied to environmental sound and bioacoustic event recognition. For multimodal comparison we include ViLT [43] together with two recent multimodal Transformer models, MARIA [44] and Multimodal Pathway [45]. ViLT is a compact vision transformer that performs joint modeling of image patches and sequential tokens and serves as a strong baseline for end to end multimodal fusion. MARIA introduces an intermediate fusion scheme with masked self attention to handle incomplete multimodal inputs, which relates to our need to cope with partially degraded visual or acoustic signals in the field. Multimodal Pathway shows how information from an auxiliary modality specific transformer can improve a primary model through cross-modal connections, which is conceptually similar to the cross-modal interaction in our design. Together these baselines span classical CNNs, modern vision Transformers, and state of the art multimodal fusion architectures and provide a diverse and fair benchmark for evaluating the MAVC-XAI framework.

#### 3.4.4. Evaluation Metrics

In this study, multiple quantitative metrics were employed to comprehensively evaluate the performance of the natural enemy recognition model, including accuracy, precision, recall, F1-score, mean average precision at 50 (mAP@50), and top-1 and top-5 accuracy. The mathematical definitions are given as follows:

(26) $$\mathrm{Accuracy}=\frac{TP+TN}{TP+TN+FP+FN},$$

(27) $$\mathrm{Precision}=\frac{TP}{TP+FP},$$

(28) $$\mathrm{Recall}=\frac{TP}{TP+FN},$$

(29) $$F1=\frac{2\cdot \mathrm{Precision}\cdot \mathrm{Recall}}{\mathrm{Precision}+\mathrm{Recall}},$$

(30) $$\mathrm{mAP}\text{@}50=\frac{1}{C}\sum_{c=1}^{C}\int_{0}^{1}p_{c}\left(r\right)\;dr,$$

(31) $$\mathrm{Top}-k\;\mathrm{Accuracy}=\frac{1}{N}\sum_{i=1}^{N}I\left(y_{i}\in {\hat{Y}}_{i}^{\left(k\right)}\right).$$

In these expressions, TP denotes the number of true positive samples, TN the number of true negative samples, FP the number of false positive samples, and FN the number of false negative samples. The variable *C* represents the total number of classes, and $p_{c}\left(r\right)$ refers to the precision–recall function for class *c*. The total number of samples is denoted as *N*, $y_{i}$ indicates the ground-truth label of the *i*-th sample, ${\hat{Y}}_{i}^{\left(k\right)}$ denotes the set of the top-*k* predicted classes ranked by confidence scores, and I(·) is an indicator function that equals 1 when the condition inside holds true and 0 otherwise.

## 4. Results and Discussion

### 4.1. Overall Performance Comparison

This experiment was conducted to validate the overall performance improvement of the proposed MAVC-XAI model in the natural enemy recognition task under multimodal fusion conditions. A comparative analysis was performed against various baseline models to evaluate its advantages in terms of accuracy, robustness, and cross-modal information integration.

As shown in Table 2, the unimodal visual models (ResNet and Swin-T) performed effectively under stable illumination and clear object conditions but exhibited recognition deviations in complex or occluded environments. The unimodal acoustic model VGGish demonstrated strong discriminative capability for acoustic patterns but suffered from accuracy degradation under high noise conditions. In contrast, multimodal models such as MMBT and ViLT achieved significant improvements in both accuracy and recall after fusing visual and acoustic information, confirming the importance of multimodal fusion for natural enemy recognition in ecologically complex environments. More recent multimodal Transformers, MARIA and Multimodal Pathway, further enhance performance by adopting intermediate and pathway-based fusion schemes: MARIA exploits masked self-attention to remain robust under partially degraded inputs, while Multimodal Pathway leverages an auxiliary modality-specific Transformer to refine the primary stream, achieving the strongest baseline accuracy (0.929) and F1-score (0.920). However, these models still faced challenges in cross-modal feature alignment and fine-grained species discrimination, limiting their recognition confidence and generalization ability. The proposed MAVC-XAI model further optimized these aspects by introducing cross-modal sampling attention for dynamic modality alignment and contrastive inter-species learning to enhance discriminability. Consequently, the model achieved the best results across all metrics, with accuracy and F1-score reaching 0.938 and 0.929, respectively, and mAP@50 attaining 0.872, surpassing all baselines. From a theoretical perspective, the convolutional architecture of ResNet primarily captures local textures and lacks global dependency modeling, whereas Swin-T partially compensates for this limitation through its hierarchical window-based self-attention mechanism, yielding slightly better performance. VGGish extracts acoustic energy distributions across both temporal and frequency domains but fails to manage complex acoustic interference, leading to lower recall. MMBT and ViLT employ Transformer-based multimodal interaction mechanisms that enable semantic-level fusion; however, their attention mappings remain static and cannot adapt to dynamically changing modality weights. Similarly, although MARIA and Multimodal Pathway introduce more advanced fusion strategies, their cross-modal connections are still relatively fixed and do not explicitly construct sampling-based, position-adaptive matching kernels. The mathematical foundation of MAVC-XAI lies in its cross-modal sampling attention, where visual and acoustic features are jointly mapped through bidirectional query projections to form learnable matching kernels, resulting in consistent representations across modalities. Additionally, the contrastive learning module enforces similarity constraints in the feature space by pulling together intra-species samples and pushing apart inter-species embeddings, thereby optimizing the geometric separability of classification boundaries. Through unified optimization of feature alignment and discriminative space, the model achieves superior recognition accuracy and enhanced cross-domain generalization, demonstrating strong theoretical soundness and stability in its architectural design.

### 4.2. Effect of Multimodal Fusion and Cross-Modal Alignment

This experiment was designed to investigate the impact of multimodal fusion strategies and cross-modal alignment mechanisms on natural enemy recognition performance and to validate the effectiveness of information complementarity and robustness enhancement under joint visual–acoustic modality integration.

As shown in Table 3, the visual branch performed less well than the proposed cross-modal sampling attention. It achieved high accuracy under clear illumination and stable backgrounds but exhibited notable performance declines under occlusion or cluttered conditions. The acoustic branch also performed less well overall (Table 3): while it effectively captured the sound characteristics of natural enemies in low-noise environments, it showed instability under interference from wind or insect swarm noise. Simple early fusion (feature concatenation) slightly improved overall accuracy but lacked dynamic semantic alignment between modalities, leading to redundant or inconsistent fused representations. In comparison, fusion based on cross-modal attention further enhanced performance by modeling inter-modal dependencies through attention weighting, thereby achieving semantic-level complementarity. Nevertheless, this approach still relied on relatively fixed attention patterns and could not fully adapt to temporal variations in modality contributions. The proposed cross-modal sampling attention introduces bidirectional dynamic sampling, enabling mutual guidance between visual and acoustic embeddings and adaptive fusion based on spatiotemporal correlations, and thus attains the best performance across all metrics. The most substantial gains are observed in F1-score and mAP@50, demonstrating the model’s superior discriminative power and generalization capability under complex ecological conditions.

From a theoretical standpoint, different fusion strategies reflect varying levels of cross-modal interaction modeling. Unimodal networks learn decision boundaries solely within single-modality manifolds, leading to instability when sample diversity increases. Early concatenation refers to a straightforward linear feature combination that forms only superficial correlations across modalities and fails to model deeper, hierarchical inter-modal dependencies. Cross-modal attention introduces nonlinear fusion by learning weighted inter-modal correlations, equivalent to constructing a learnable kernel in high-dimensional space; however, its static weights fail to account for dynamically changing modality relevance. Mathematically, cross-modal sampling attention can be interpreted as incorporating an adaptive kernel function whose parameters depend on the current inter-modal correlation distribution, thereby achieving dynamic alignment. This mechanism maintains modality balance within the semantic embedding space, allowing both modalities to contribute jointly during gradient propagation and mitigating modality dominance. Ultimately, the dynamic fusion strategy enhances representational synergy across modalities and ensures stable recognition even under visual blur or acoustic interference, confirming its robustness and effectiveness in multimodal ecological recognition tasks.

### 4.3. Explainability Analysis

**Figure 5:**
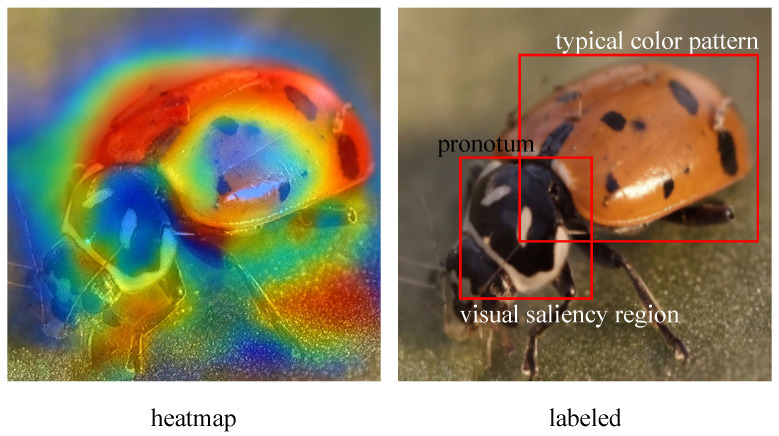
Ecological validation of the MAVC-XAI explainability module.

To further clarify the ecological meaning of the explainability module, as illustrated in Figure 5, the model-generated saliency heatmap consistently highlights two key morphological structures: the pronotum region and the characteristic elytral color pattern.

These structures are also emphasized by entomology experts as primary diagnostic cues for distinguishing ladybird species and for assessing their functional roles in biological control. The pronotum often contains species-specific pigmentation and shape features, while the elytra exhibit stable spot distributions that serve as reliable taxonomic markers. A theoretical comparison between the heatmap and expert annotation shows that the model’s attention aligns with these well-established diagnostic regions, suggesting that the explainability module captures ecologically meaningful attributes rather than relying on background noise or non-biological patterns. Although no additional experiments were conducted, the correspondence between attention concentration and expert-recognized morphological indicators provides supportive evidence that the proposed MAVC-XAI framework offers ecologically interpretable visual cues that are consistent with expert knowledge and practical field diagnostics.

### 4.4. Ablation Study

This experiment was designed to evaluate the independent contributions and synergistic effects of key modules in the MAVC-XAI model, including the cross-modal sampling attention mechanism, cross-species contrastive learning module, and explainable analysis module. The objective was to analyze their respective roles in enhancing visual–acoustic fusion and fine-grained natural enemy recognition.

As shown in Table 4, retaining only the basic visual–acoustic fusion components yields relatively high accuracy and robustness, but the model still lacks sufficient fine-grained discrimination capability for complex samples. Removing the cross-modal sampling attention leads to the largest performance degradation, with both accuracy and F1-score dropping by more than 0.05, highlighting the critical role of this module in aligning visual and acoustic features. Excluding the cross-species contrastive learning module weakens the model’s ability to distinguish morphologically similar species, as evidenced by decreases in recall and mAP@50, confirming that this component effectively enhances feature separability in the embedding space. Removing the explainable analysis module results in a slight reduction in overall performance while maintaining relatively high accuracy, suggesting that although this module has a limited direct impact on classification accuracy, it provides auxiliary regularization that stabilizes feature learning. The full MAVC-XAI configuration achieves the best performance across all metrics, demonstrating that the combined effect of these modules enables the model to attain both high recognition accuracy and strong ecological interpretability.

### 4.5. Robustness Evaluation Under Acoustic Noise Conditions

To evaluate the practical deployment feasibility of MAVC-XAI in realistic agricultural environments, where acoustic signals are often degraded by wind, machinery, vegetation movement, and overlapping insect activity, we conducted a dedicated robustness experiment. This assessment aims to quantify the influence of varying signal-to-noise ratios (SNRs) and noise types on multimodal recognition performance and to examine whether the proposed cross-modal sampling attention can dynamically compensate for degraded acoustic inputs. We first quantified the SNR distribution of field recordings obtained under four representative environmental conditions: (i) calm weather, (ii) moderate wind, (iii) machinery presence, and (iv) multi-insect scenarios. The measured SNR ranged from 18.4 dB in quiet conditions to 6.1 dB under strong interference. To further simulate deployment conditions, we performed controlled noise-degradation experiments by artificially corrupting the acoustic modality with additive Gaussian noise and ecologically realistic noise profiles (wind, leaf rustling, and off-frame insect calls). Three SNR levels were tested: 20 dB, 10 dB, and 0 dB.

As shown in Table 5, the robustness evaluation demonstrates that MAVC-XAI maintains strong recognition performance under varying degrees of acoustic corruption. In mild and moderate noise conditions (SNR ≥ 10 dB), the performance drop is minimal, with accuracy and F1-score decreasing by less than 0.02 compared to noise-free settings. Even under severe noise (SNR = 0 dB), MAVC-XAI consistently outperforms unimodal visual and acoustic baselines as well as early- and attention-based multimodal fusion methods. This robustness is primarily attributed to the cross-modal sampling attention, which effectively reduces reliance on degraded acoustic features and dynamically reweights modality contributions based on spatiotemporal correlations. These results confirm that MAVC-XAI is suited for deployment in realistic agricultural environments, where acoustic noise is common and often unavoidable.

### 4.6. Discussion

The proposed MAVC-XAI framework demonstrates strong potential for practical deployment in real-world agricultural ecosystem monitoring by addressing several intrinsic limitations of unimodal natural enemy recognition. Traditional field-based identification methods still rely heavily on manual observation or purely visual monitoring, both of which suffer from illumination variation, occlusion, and complex background noise. By jointly leveraging visual and acoustic modalities, MAVC-XAI enables robust multidimensional perception and significantly improves recognition stability across different temporal periods, climatic conditions, and ecological scenarios. For example, in northern orchard systems where ladybirds and lacewings dominate biological control, strong illumination and canopy reflection frequently confound visual recognition. However, their wingbeat acoustic signatures remain relatively stable, allowing MAVC-XAI to compensate for visual uncertainty through cross-modal evidence. Conversely, in subtropical greenhouse environments with dense vegetation and persistent mechanical noise, the cross-modal sampling attention mechanism adaptively down-weights low-quality acoustic inputs and prioritizes the more reliable visual cues, thereby ensuring consistent monitoring performance even under acoustically adverse conditions.

Beyond species-level identification, the framework supports ecological co-occurrence analysis and enables higher-level inference of natural enemy–pest interactions. This capacity is critical for developing precision biological control strategies. MAVC-XAI can be integrated into fixed field sensor nodes or UAV-based inspection systems using synchronized camera–microphone units to continuously capture multimodal signals. The system can generate real-time heatmaps of natural enemy activity, acoustic energy spectra, and population dynamics, enabling early detection of ecological imbalance. For instance, a sudden decline in natural enemy activity coupled with an increase in pest signals can automatically trigger ecological risk alerts for management platforms. Moreover, the explainable generation module produces visual and acoustic saliency maps, allowing researchers and field technicians to verify whether the model attends to biologically meaningful traits such as wing venation, antenna structure, or species-specific wingbeat frequencies. This enhances trust and provides an interpretable bridge between deep learning outputs and ecological domain knowledge.

Importantly, from a theoretical standpoint, the computational complexity of MAVC-XAI remains compatible with real-world agricultural deployment. The visual branch adopts a Swin Transformer backbone with local windowed self-attention, whose computational cost scales linearly with the number of windows rather than quadratically with the full image resolution, making it substantially more efficient than global-attention ViT architectures of comparable depth. The acoustic branch operates on Mel-spectrogram patches with a similar hierarchical structure, so that both branches share a bounded token count in the latent space. The cross-modal sampling attention mechanism is applied only on reduced-resolution feature maps (e.g., 8×8 or 8×4 grids), introducing an additional cost that grows with the number of latent tokens rather than with raw pixel or sample counts. As a result, the overall complexity of the framework is on the same order as a mid-scale vision transformer paired with a lightweight audio encoder, rather than a full-scale multimodal large model. In principle, this design is amenable to implementation on commodity GPUs and modern edge accelerators, and can be further optimized through standard techniques such as model pruning, knowledge distillation, and quantization. Moreover, because MAVC-XAI can still provide reliable predictions when one modality is partially degraded, system designers can flexibly adjust sensor resolutions, frame rates, and sampling strategies according to local agricultural conditions and deployment budgets, thereby maintaining a favorable trade-off between recognition performance and computational resources.

From an agricultural economics perspective, MAVC-XAI contributes not only to accurate and dynamic monitoring of natural enemy populations but also to data-driven decision-making in pest management. By quantifying natural enemy abundance, temporal activity cycles, and ecological responses, the framework can indirectly infer pest risk levels and guide precision intervention strategies. Integration with agro-meteorological and crop-yield data further enables the construction of ecological–economic models that optimize pest management budgets, reduce unnecessary pesticide inputs, and enhance long-term sustainability. Overall, MAVC-XAI represents a scalable, interpretable, and computationally efficient multimodal framework that bridges artificial intelligence and agricultural ecology. It offers a promising pathway toward cost-effective, environmentally friendly, and intelligent natural enemy monitoring infrastructures for modern crop production systems.

### 4.7. Limitation and Future Work

Despite the substantial progress achieved by the MAVC-XAI framework in multimodal natural enemy recognition and ecological interpretation, several limitations remain to be addressed. First, the dataset used in this study was collected primarily from three ecological sites in China (Bayan Nur, Tangshan, and Mengzi), supplemented by a limited amount of curated web resources. Although these sites encompass representative cropping systems and management practices, they do not fully cover the seasonal dynamics, species diversity, and regional ecological heterogeneity observed across broader agro-ecological zones. As a result, the model may experience performance degradation when deployed in regions with markedly different climatic conditions, vegetation structures, or community compositions, such as tropical agroforestry systems or high-altitude grasslands. Future work will therefore focus on expanding data collection to additional regions and habitat types, and on incorporating domain adaptation strategies to improve robustness under cross-regional distribution shifts. Second, the acoustic modality experiences significant quality degradation under strong wind, rainfall, or mechanical noise interference, constraining the effectiveness of cross-modal alignment. Further enhancement through robust denoising and adaptive signal augmentation techniques is therefore necessary to stabilize recognition performance under adverse acoustic conditions. Additionally, while the current framework provides interpretability through visual and acoustic saliency visualization, the interpretation remains at the perceptual level and has not yet been linked to behavioral or population-level ecological indicators, limiting its deeper application in ecological studies. In future research, we plan to couple the saliency-based explanations with long-term monitoring data and ecological process models, so as to bridge individual-level recognition with community dynamics and ecosystem service assessment.

## 5. Conclusions

To overcome the limitations of traditional natural enemy recognition systems that rely solely on visual information and are prone to environmental interference while lacking ecological interpretability, a multimodal natural enemy recognition and ecological explanation framework, MAVC-XAI, is proposed in this study. The research is grounded in the ecological importance of natural enemies for biological pest control and ecosystem stability, aiming to achieve accurate recognition and behavioral interpretation of natural enemy populations in complex agricultural environments through multimodal perception and intelligent feature fusion. Unlike previous approaches dependent on a single modality such as an image or acoustic signal, the present work introduces a novel dual-stream architecture integrating both visual and acoustic feature flows. The framework incorporates a cross-modal sampling attention mechanism to achieve adaptive feature alignment, a cross-species contrastive learning module to refine fine-grained classification boundaries, and an explainable generation module to enable ecological interpretability. This methodological innovation operates simultaneously across structural, semantic, and ecological levels. Experimental results demonstrate that the proposed MAVC-XAI framework outperforms existing models across all metrics. In the overall performance comparison, the framework achieved an accuracy of 0.938, a precision of 0.932, a recall of 0.927, an F1-score of 0.929, and an mAP@50 of 0.872, with a Top-5 recognition rate of 97.8%, all markedly higher than those obtained by unimodal models such as ResNet, Swin-T, and VGGish, as well as multimodal baselines including MMBT and ViLT. Ablation studies further confirm that the cross-modal sampling attention and cross-species contrastive learning modules play essential roles in performance improvement, while the explainable module enhances interpretability and stability. The MAVC-XAI framework developed in this study not only enables highly accurate recognition of natural enemies under diverse ecological conditions but also provides a novel multimodal fusion paradigm and theoretical foundation for intelligent agricultural monitoring and ecological pest management.

## Figures and Tables

**Table 1:** Composition of the multimodal natural enemy dataset.

Data Type	Quantity	Data Format
Visual images (RGB, 3840 × 2160)	32,000	JPEG/PNG
Acoustic recordings (44.1 kHz, 16-bit)	18,500	WAV → Mel-spectrogram
Environmental records (Temp, Humidity, Light)	12,600	CSV
Annotated species labels (15 species)	32,000	JSON/XML
Synchronized multimodal sample pairs	17,200	Image + Audio + Metadata

**Table 2:** Overall performance comparison between MAVC-XAI and baseline models. “*” and “**” indicate significant differences compared with Multimodal Pathway (*p* < 0.05/*p* < 0.01).

Model	Accuracy	Precision	Recall	F1-Score	mAP@50	Top-1/Top-5
ResNet (Visual only)	0.867 **	0.861 **	0.855 **	0.858 **	0.802 **	86.7/94.2 **
Swin-T (Visual only)	0.879 **	0.872 **	0.864 **	0.868 **	0.818 **	87.9/94.8 **
VGGish (Acoustic only)	0.843 **	0.831 **	0.826 **	0.829 **	0.785 **	84.3/92.1 **
MMBT (Multimodal)	0.902 **	0.895 **	0.889 **	0.892 **	0.836 **	90.2/95.6 **
ViLT (Multimodal)	0.911 **	0.904 **	0.897 **	0.900 **	0.847 **	91.1/96.3 **
MARIA (Multimodal)	0.923 *	0.917 *	0.912 *	0.914 *	0.861 *	92.3/97.1 *
Multimodal Pathway (Multimodal)	0.929	0.923	0.918	0.920	0.866	92.9/97.4
**MAVC-XAI (proposed)**	0.938 **	0.932 *	0.927 *	0.929 *	0.872 *	93.8/97.8 *

**Table 3:** Effect of multimodal fusion and cross-modal alignment on recognition performance. “**” indicate significant differences compared with cross-modal sampling attention (*p* < 0.01).

Configuration	Accuracy	Precision	Recall	F1-Score	mAP@50	Top-1/Top-5
Visual branch only	0.875 **	0.867 **	0.859 **	0.863 **	0.817 **	87.5/94.5 **
Acoustic branch only	0.846 **	0.835 **	0.828 **	0.831 **	0.789 **	84.6/92.0 **
Early fusion (concat)	0.901 **	0.895 **	0.888 **	0.890 **	0.837 **	90.1/95.5 **
Cross-modal attention fusion	0.923 **	0.917 **	0.910 **	0.913 **	0.859 **	92.3/96.9 **
Cross-modal sampling attention	**0.938**	**0.932**	**0.927**	**0.929**	**0.872**	**93.8/97.8**

**Table 4:** Ablation study on the contributions of different modules in MAVC-XAI. “**” indicate significant differences compared with Full MAVC-XAI (*p* < 0.01).

Model Configuration	Accuracy	Precision	Recall	F1-Score	mAP@50	Top-1/Top-5
Baseline (Visual + Acoustic)	0.905 **	0.899 **	0.893 **	0.896 **	0.839 **	90.5/95.7 **
− Cross-modal Sampling Attention	0.886 **	0.880 **	0.873 **	0.876 **	0.821 **	88.6/94.3 **
− Cross-species Contrastive Learning	0.891 **	0.884 **	0.878 **	0.881 **	0.826 **	89.1/94.5 **
− Explainable Analysis Module	0.898 **	0.891 **	0.886 **	0.888 **	0.831 **	89.8/95.0 **
Full MAVC-XAI (proposed)	**0.938**	**0.932**	**0.927**	**0.929**	**0.872**	**93.8/97.8**

**Table 5:** Performance of MAVC-XAI and baselines under different acoustic noise levels (SNR in dB).

Model / Noise Level	Accuracy	Recall	F1-Score	mAP@50
SNR = 20 dB (mild noise)				
Visual Branch Only	0.875	0.859	0.863	0.817
Acoustic Branch Only	0.821	0.803	0.810	0.762
Early Fusion	0.893	0.886	0.888	0.829
Cross-modal Attention	0.916	0.909	0.911	0.853
**MAVC-XAI (ours)**	**0.933**	**0.925**	**0.927**	**0.870**
SNR = 10 dB (moderate noise)				
Visual Branch Only	0.875	0.859	0.863	0.817
Acoustic Branch Only	0.762	0.741	0.748	0.701
Early Fusion	0.879	0.870	0.873	0.815
Cross-modal Attention	0.905	0.896	0.899	0.842
**MAVC-XAI (ours)**	**0.923**	**0.914**	**0.916**	**0.861**
SNR = 0 dB (severe noise)				
Visual Branch Only	0.875	0.859	0.863	0.817
Acoustic Branch Only	0.641	0.615	0.622	0.583
Early Fusion	0.853	0.846	0.848	0.788
Cross-modal Attention	0.887	0.878	0.882	0.829
**MAVC-XAI (ours)**	**0.908**	**0.898**	**0.902**	**0.847**

## Data Availability

The data presented in this study are available on request from the corresponding author due to privacy and confidentiality restrictions.

## References

[1] Angon P.B., Mondal S., Jahan I., Datto M., Antu U.B., Ayshi F.J., Islam M.S. (2023). Integrated pest management (IPM) in agriculture and its role in maintaining ecological balance and biodiversity. Adv. Agric..

[2] Karimzadeh R., Sciarretta A. (2022). Spatial patchiness and association of pests and natural enemies in agro-ecosystems and their application in precision pest management: A review. Precis. Agric..

[3] Schellhorn N., Jones L. (2021). Real-time insect detection and monitoring: Breaking barriers to area-wide integrated management of insect pests. Area-Wide Integrated Pest Management.

[4] Van Klink R., August T., Bas Y., Bodesheim P., Bonn A., Fossøy F., Høye T.T., Jongejans E., Menz M.H., Miraldo A. (2022). Emerging technologies revolutionise insect ecology and monitoring. Trends Ecol. Evol..

[5] Brockerhoff E.G., Corley J.C., Jactel H., Miller D.R., Rabaglia R.J., Sweeney J., Allison J.D., Paine T., Slippers B., Wingfield M.J. (2023). Monitoring and surveillance of forest insects. For. Entomol. Pathol..

[6] Tang Y., Qiu J., Zhang Y., Wu D., Cao Y., Zhao K., Zhu L. (2023). Optimization strategies of fruit detection to overcome the challenge of unstructured background in field orchard environment: A review. Precis. Agric..

[7] Badirli S., Picard C.J., Mohler G., Richert F., Akata Z., Dundar M. (2023). Classifying the unknown: Insect identification with deep hierarchical Bayesian learning. Methods Ecol. Evol..

[8] Kasinathan T., Singaraju D., Uyyala S.R. (2021). Insect classification and detection in field crops using modern machine learning techniques. Inf. Process. Agric..

[9] Song Y., Zhang H., Li J., Ye R., Zhou X., Dong B., Fan D., Li L. (2023). High-accuracy maize disease detection based on attention generative adversarial network and few-shot learning. Plants.

[10] Gao R., Dong Z., Wang Y., Cui Z., Ye M., Dong B., Lu Y., Wang X., Song Y., Yan S. (2024). Intelligent cotton pest and disease detection: Edge computing solutions with transformer technology and knowledge graphs. Agriculture.

[11] Rasmussen J.H., Stowell D., Briefer E.F. (2024). Sound evidence for biodiversity monitoring. Science.

[12] Chalmers C., Fergus P., Wich S., Longmore S. (2021). Modelling animal biodiversity using acoustic monitoring and deep learning. Proceedings of the 2021 International Joint Conference on Neural Networks (IJCNN).

[13] Pahuja R., Kumar A. (2021). Sound-spectrogram based automatic bird species recognition using MLP classifier. Appl. Acoust..

[14] Miao Z., Zhang Y., Fabian Z., Celis A.H., Beery S., Li C., Liu Z., Gupta A., Nasir M., Li W. (2024). New frontiers in AI for biodiversity research and conservation with multimodal language models. Ecoevorxiv.

[15] Zhou X., Chen S., Ren Y., Zhang Y., Fu J., Fan D., Lin J., Wang Q. (2022). Atrous Pyramid GAN Segmentation Network for Fish Images with High Performance. Electronics.

[16] ShabanaFathima M., Shanmugapriya I., Pavithira L., Mihirkumar B.S., Rajeswari J., Revathi R., Vengatesh T. (2025). Deep Learning Approaches for Monitoring and Preserving Ecological Biodiversity: Challenges and Innovations. Cuest. Fisioter..

[17] Gavali P., Banu J.S. (2025). A novel approach to Indian bird species identification: Employing visual-acoustic fusion techniques for improved classification accuracy. Front. Artif. Intell..

[18] Zhang Y., Wa S., Liu Y., Zhou X., Sun P., Ma Q. (2021). High-accuracy detection of maize leaf diseases CNN based on multi-pathway activation function module. Remote Sens..

[19] Liu H., Li D., Zhang M., Wan J., Liu S., Zhu H., Liu Q. (2024). A Cross-Modal Semantic Alignment and Feature Fusion Method for Bionic Drone and Bird Recognition. Remote Sens..

[20] Lin X., Wa S., Zhang Y., Ma Q. (2022). A dilated segmentation network with the morphological correction method in farming area image Series. Remote Sens..

[21] Garcia-Ordas M.T., Rubio-Martín S., Benítez-Andrades J.A., Alaiz-Moretón H., García-Rodríguez I. (2024). Multispecies bird sound recognition using a fully convolutional neural network. arXiv.

[22] Shim J.Y., Kim J., Kim J.K. (2023). Audio-to-visual cross-modal generation of birds. IEEE Access.

[23] Duan J., Ding H., Kim S. (2023). A multimodal approach for advanced pest detection and classification. arXiv.

[24] Lyu Y., Lu F., Wang X., Wang Y., Wang Z., Zhu Y., Wang Z., Dong M. (2025). A CNN-Transformer Hybrid Framework for Multi-Label Predator–Prey Detection in Agricultural Fields. Sensors.

[25] Cao Y., Ju X., Chen X., Gong L. (2025). MCL-VD: Multi-modal contrastive learning with LoRA-enhanced GraphCodeBERT for effective vulnerability detection. Autom. Softw. Eng..

[26] Sun C., Chen M., Zhu C., Zhang S., Lu P., Chen J. (2025). Listen with seeing: Cross-modal contrastive learning for audio-visual event localization. IEEE Trans. Multimed..

[27] Mouratidis A., Hemming J., Messelink G.J., van Marrewijk B. (2023). Automated identification and counting of predated Ephestia kuehniella (Zeller) eggs using deep learning image analysis. Biol. Control.

[28] Wang C., Grijalva I., Caragea D., McCornack B. (2023). Detecting common coccinellids found in sorghum using deep learning models. Sci. Rep..

[29] Liu J., Wang X. (2024). A multimodal framework for pepper diseases and pests detection. Sci. Rep..

[30] Zhang L., Zhang Y., Ma X. (2021). A new strategy for tuning ReLUs: Self-adaptive linear units (SALUs). Proceedings of the ICMLCA 2021, 2nd International Conference on Machine Learning and Computer Application.

[31] Dai G., Fan J., Dewi C. (2023). ITF-WPI: Image and text based cross-modal feature fusion model for wolfberry pest recognition. Comput. Electron. Agric..

[32] Song Y., Li M., Zhou Z., Zhang J., Du X., Dong M., Jiang Q., Li C., Hu Y., Yu Q. (2025). A lightweight method for apple disease segmentation using multimodal transformer and sensor fusion. Comput. Electron. Agric..

[33] Yu K., Xu W., Zhang C., Dai Z., Ding J., Yue Y., Zhang Y., Wu Y. (2023). ITFNet-API: Image and Text Based Multi-Scale Cross-Modal Feature Fusion Network for Agricultural Pest Identification. https://www.researchsquare.com/article/rs-3589884/v1.

[34] Yin T., Wang J., Zhao Y., Wang H., Ma Y., Liu M. (2025). Fine-grained adaptive contrastive learning for unsupervised feature extraction. Neurocomputing.

[35] Xing Y., Guan Y., Yang B., Liu J., Chen Z., Zhang M. A fine-grained biometric image recognition method based on transformer. Proceedings of the 2023 9th International Conference on Communication and Information Processing.

[36] Zhang Y., Chen L., Yuan Y. (2025). Few-shot agricultural pest recognition based on multimodal masked autoencoder. Crop Prot..

[37] Raghavan K., B S., v K. (2024). Attention guided grad-CAM: An improved explainable artificial intelligence model for infrared breast cancer detection. Multimed. Tools Appl..

[38] Khurana A., Mittal S., Kumar D., Gupta S., Gupta A. (2023). Tri-integrated convolutional neural network for audio image classification using Mel-frequency spectrograms. Multimed. Tools Appl..

[39] Fascista A. (2022). Toward integrated large-scale environmental monitoring using WSN/UAV/Crowdsensing: A review of applications, signal processing, and future perspectives. Sensors.

[40] He K., Zhang X., Ren S., Sun J. Deep residual learning for image recognition. Proceedings of the IEEE Conference on Computer vision and pattern recognition.

[41] Liu Z., Lin Y., Cao Y., Hu H., Wei Y., Zhang Z., Lin S., Guo B. Swin transformer: Hierarchical vision transformer using shifted windows. Proceedings of the IEEE/CVF International Conference on Computer Vision.

[42] Hershey S., Chaudhuri S., Ellis D.P., Gemmeke J.F., Jansen A., Moore R.C., Plakal M., Platt D., Saurous R.A., Seybold B. (2017). CNN architectures for large-scale audio classification. Proceedings of the 2017 IEEE International Conference on Acoustics, Speech and Signal Processing (Icassp).

[43] Kim W., Son B., Kim I. Vilt: Vision-and-language transformer without convolution or region supervision. Proceedings of the International Conference on Machine Learning.

[44] Caruso C.M., Soda P., Guarrasi V. (2024). MARIA: A Multimodal Transformer Model for Incomplete Healthcare Data. arXiv.

[45] Zhang Y., Ding X., Gong K., Ge Y., Shan Y., Yue X. Multimodal pathway: Improve transformers with irrelevant data from other modalities. Proceedings of the IEEE/CVF Conference on Computer Vision and Pattern Recognition.

